# Natural and Artificial Aging Effects on the Deformation Behaviors of Al–Mg–Zn Alloy Sheets

**DOI:** 10.3390/ma17184478

**Published:** 2024-09-12

**Authors:** Kwangmin Choi, Sangjun Lee, Donghyun Bae

**Affiliations:** 1 Department of Materials Science and Engineering, Yonsei University, Seoul 03722, Republic of Korea; ckm88wkd@yonsei.ac.kr (K.C.);; 2 Institute of Industrial Science, The University of Tokyo, 4-6-1 Komaba, Meguro 153-8505, Tokyo, Japan

**Keywords:** aluminum alloys, artificial aging, natural aging, precipitates, dislocations, precipitate-free zone

## Abstract

This study investigated the effects of aging profiles on the precipitate formation and the corresponding strengthening and deformation behaviors of Al–Mg–Zn alloys. The alloys subjected to natural aging (NA) demonstrated significantly enhanced ductility at equivalent stress levels compared to those subjected to artificial aging (AA). In AA-treated alloys, η′ and η-phases with incoherent interfaces were formed, while GP zones and solute clusters were dominantly exhibited in the NA-treated alloy with a coherent interface with the matrix. Due to the change in interface bonding, the dislocation movement and pinning behavior after deformation are varied depending on the aging conditions of Al–Mg–Zn alloy sheet. Thus, the elongation to fracture of the NA alloy sheet was improved compared to that of the AA alloy sheet because of the enhanced work-hardening capacity and the thin precipitate-free zone (PFZ). Deformation textures and dislocation densities varied between NA and AA treatments, as revealed by electron backscatter diffraction (EBSD) and kernel average misorientation (KAM) analysis. The interactions between the precipitates, dislocations, and the PFZ in the AA- and NA-treated alloys were analyzed via transmission electron microscopy (TEM). The insights gained from this research provide a valuable foundation for industrial applications, particularly in sectors demanding lightweight, high-strength materials, where optimizing the aging process can lead to significant performance improvement and cost savings.

## 1. Introduction

The demand for high-performance, lightweight aluminum alloys increases rapidly owing to the growing environmental concerns in industries [1,2,3,4]. The 7xxx series of Al alloys comprising Al, Mg, Zn, and Cu are promising structural materials with high strength-to-weight ratios because of their exceptional precipitate strengthening. The general precipitation in an Al–Zn–Mg alloy can be described as follows [5]:

Supersaturated solid solution (SSS) → Guinier–Preston (GP) zone (GP I, GP II) → metastable phase η′ → equilibrium phase η (MgZn_2_)

In the initial stage of aging, a GP zone, which has a fully coherent interface with the matrix, is formed from the supersaturated solid solution matrix as a precursor for the nucleation of η′. Two types of precipitates (GP I and GP II) exist in the GP zone. The GP I zone consists of Al–Zn and Zn–Mg on the {001} plane of the aluminum matrix with a fully coherent interface. The GP II zone, however, is treated as a Zn cluster on the {111} plane of the aluminum matrix [6]. The metastable phase η′ is the major hardening precipitate responsible for the peak hardening of the Al–Zn–Mg–(Cu) alloy. The stable phase η (MgZn_2_) is obtained after over-aging [7].

Typically, the mechanical properties of Al–Zn–Mg alloys can be enhanced through artificial aging (AA), which is performed under “elevated temperatures” between 100 and 200 °C for most conventional aluminum alloys [8,9]. The strength of Al alloys is controlled by the type, morphology, and physical characteristics of the precipitates, which in turn depend on the composition and the additional solute elements [4,10]. A high-strength 7000 series aluminum alloy with T-phase (Mg_32_(Al, Zn)_49_) precipitates was obtained at a high Zn/Mg ratio. Strengthening precipitates can be designed by changing the Zn/Mg ratio, such as T, η′, and η-phases [9]. Zhao et al. [11] obtained Al–Mg alloy sheets with desirable mechanical properties by adding adequate Zn. Hot-rolled Al–9.2Mg–0.5Zn alloy exhibited a higher ultimate tensile strength (UTS, 434 ± 5 MPa), yield strength (YS, 228 ± 2 MPa), and elongation (48 ± 2%) than Al–Mg alloy sheets. Smith and Grant [12] observed that the precipitation rate at low-temperature aging conditions could be increased in the presence of 0.5 wt. % of the Cu element because numerous nucleated GP zones induced considerable strengthening. Liu et al. [13] reported that Ag improved the tensile properties and fracture toughness of novel Al–Mg–Zn alloy sheets by precipitation hardening.

Natural aging (NA) performed at “room temperature” also strengthens the alloy. NA leads to a different type of precipitate from AA, usually requires a long aging time, and achieves a lower hardness level. Liu et al. [14] investigated the formation of solute clusters and GP zones in Al–Mg–Zn alloys during NA. Furthermore, the formation of clusters in long-term natural aging is also defined. Geng et al. [15] studied the effects of Cu atoms on the precipitation and solute clustering during the initial stages of NA. Cu atoms promoted the formation of solute clusters, so a large number of clusters influenced the transformation of T′-phases. Furthermore, the positive effect of NA on the mechanical properties of Al–Mg–Zn alloy was investigated. Zhao et al. [16] explored the influence of a four-stage aging treatment on the strength and elongation of 7000 series of aluminum alloy sheets after AA and NA. Before the re-aging treatment, NA enhanced the tensile strength and elongation of the alloy because of the formation of GP zones.

So far, most research on the aging of Al–Zn–Mg alloys has focused on strategies to enhance their strength or investigations about the formation mechanisms of hardening precipitates. Previously, some researchers tried to develop a ductility-enhanced Al–Mg–Zn alloy. A high-ductility Al–Zn–Mg alloy was obtained from a Zn-modified Al–Mg alloy by enhancing the work hardening [11]. Al–Mg alloy with a moderate Zn content can achieve random grain orientation and effective work hardening, leading to high ductility in hot-rolled samples. Furthermore, Mn and Ag are excellent solute elements because they can promote grain refinement to form a narrow precipitate-free zone (PFZ) in Al–Zn–Mg alloys and enhance ductility [17]. Ogura et al. used Ag-containing intermetallic compounds to stabilize grain structures and accelerate the nucleation of strengthening dispersoids. Although a few studies have investigated the methods to develop high-ductility Al–Mg–Zn alloys using extra solute elements, the specific influence of precipitates on deformation behaviors, such as texture and dislocation interactions, remains unclear. Moreover, the attempts to enhance the ductility of Al–Zn–Mg alloys and define the difference in deformation behavior in artificially and naturally aged alloys have been insufficiently reported.

In this study, the plastic deformation behaviors of Al alloys under AA and NA treatments were investigated to explore the mechanism of strength and ductility enhancement, providing insights into their specific applications. According to the aging conditions, the microstructure of the alloy is varied, and it offers an opportunity to change the dislocation activity. This study also suggests that the presence of precipitates contributes significantly to the mechanical properties with respect to texture and plastic anisotropy, which is a novel perspective to understand the structure–performance relationship of Al alloys.

## 2. Materials and Methods

Al–Mg–Zn alloys were fabricated using a conventional casting method, an electric resistivity furnace, and a permanent steel mold. Pure Al (purity 3N, Hanjin Metal) was melted at 730 °C under ambient conditions, and then Mg (Purity 4N, Hanjin Metal) and Zn (Purity 4N, Hanjin Metal) were added according to the compositions listed in Table 1. The liquid alloy was held for 30 min to achieve compositional uniformity to the alloy melts, and the melts were poured into a steel mold. Bar-type ingots were subjected to heat treatment at 500 °C for 12 h for homogenization. The surfaces of the ingots were then mechanically milled to remove surface defects, such as the oxide layer. Subsequently, hot rolling was applied to reduce the thickness of the ingot to 4 mm at a 20% reduction rate. Cold rolling was then performed at a reduction rate of 10–20% to reach a 1.0–1.2 mm thickness. The as-rolled alloy sheet was solution-heat-treated at 500 °C for 15 min to recrystallize the grain and solutionize the alloying element. Subsequently, AA and NA were conducted to enhance the mechanical properties, particularly the strength. AA was performed under 120 °C, and NA was conducted at room temperature (25 °C).

Vickers hardness and uniaxial tension tests were performed on the alloy sheets at regular time intervals. The specimens were artificially and naturally aged, then mechanically polished to remove the oxide layer. Then, the Vickers hardness test was performed with a 200 gf compression load; furthermore, analysis was performed as uniformly as possible. An Instron-type uniaxial tension test machine (RB301, R&B, Daejeon, Korea) with a constant crosshead speed (initial strain rate of 1 × 10^−3^/s) was used for the tensile tests at room temperature on a dogbone-type test sample (ASTM E8). Test specimens were prepared along various rolling directions.

The microstructures of the alloys were characterized using electron backscatter diffraction (EBSD, Oxford, UK). EBSD mapping was conducted at 20 kV and 14 mA with a 0.4 µm step size. Furthermore, the AA- and NA-treated alloy precipitates were observed using transmission electron microscopy (TEM, JEM-ARM200F(NEOARM), JEOL, Tokyo, Japan). The specimens were prepared using a focused ion beam (FIB, CrossBeam 350, Zeiss, Oberkochen, Germany).

Thermal analysis was performed using differential scanning calorimetry (DSC; Q100, TA, DE, USA). The heating rate was varied as 5, 10, 15, and 20 °C/min in an Ar gas atmosphere.

## 3. Results

### 3.1. Mechanical Properties after AA and NA

**Figure 1:**
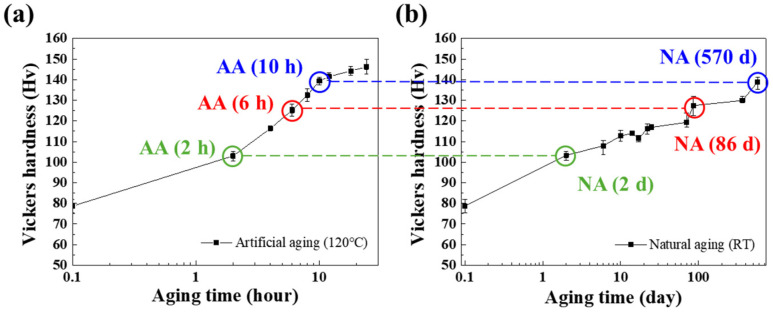
Vickers hardness as a function of aging time in (**a**) AA-treated and (**b**) NA-treated Al alloys.

The influence of AA and NA treatments on the mechanical properties of Al–Mg–Zn alloy sheets was investigated. Figure 1 presents the evolution of Vickers hardness as a function of aging time. AA (x) and NA (x) indicate the Al–Mg–Zn alloy sheets after AA and NA treatments, respectively, where x is the aging time. As time progressed, the hardness of AA samples generally increased, reaching a maximum at 24 h, as shown in Figure 1a. The increase in hardness was fast at the initial stage of AA and saturated after 12 h. In contrast, NA required a significantly longer time to reach the maximum value than AA, as shown by the dashed line in Figure 1a,b. The hardness of NA (570 d) was still lower than the maximum value of AA. Although the hardening rate at the initial stage was high for NA samples, it remained constantly lower than the AA samples. As indicated by the dashed lines and circles in Figure 1a,b, the 2, 6, and 10 h AA samples had similar hardness values to the 2-, 86-, and 570-day NA samples.

**Figure 2:**
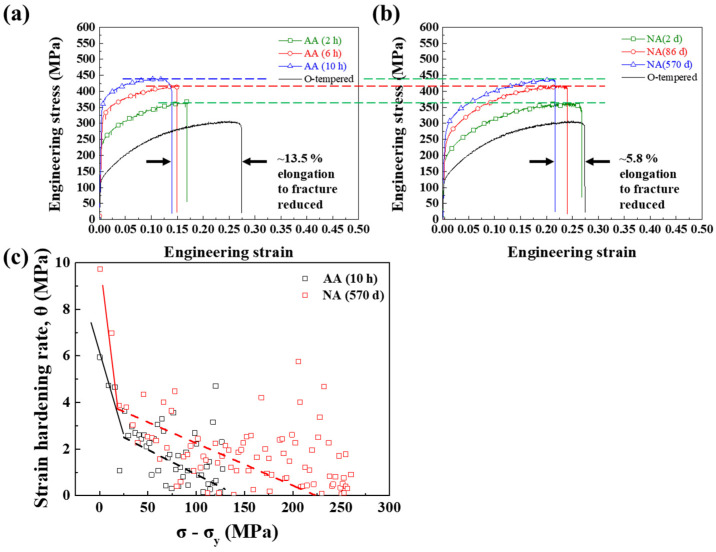
Stress–strain curves of (**a**) AA and (**b**) NA. (**c**) Graphs of instantaneous strain-hardening rate against stress increment for AA (10 h) and NA (570 d).

To investigate the difference in the mechanical properties between AA and NA samples, uniaxial tensile tests were performed on the AA (2 h) and NA (2 d), AA (6 h) and NA (86 d), and AA (10 h) and NA (570 d) parallel to the rolling direction, which showed comparable hardness levels, as marked in Figure 1a,b. As shown in Figure 2a,b with the blue, red, and green dashed lines, the UTSs of AA (2 h), AA (6 h), and AA (10 h) were similar to those of NA (2 d), NA (86 d), and NA (570 d), respectively. The hardness value is calculated by the size of the indenter after the plastic deformation; hence, the Vickers hardness value should be compared with the UTS, which represents the ability to resist deformation. This is a common relationship between Vickers hardness, yield strength, and tensile strength for aluminum alloy [18]. Though the increment trends of YS following the aging time are the same in both AA and NA samples, the value of yield strength is not similar between AA and NA. However, the AA samples showed a higher YS than the NA samples, indicating that the precipitates in the AA and NA samples contributed differently to the YS enhancement. That might have resulted from the dislocation movement during deformation; hence, the relationship between the precipitates and the strengthening behavior will be discussed later in this paper.

In contrast to the YS and UTS, the average elongation at the break of AA (~15%) was much lower than that of NA (~25%). Compared with the elongation of the solution-treated (O-tempered) alloy, the strain values decreased by approximately 5.9% and 13.6% for NA and AA, respectively. Typical aging-hardenable alloys showed a decreased ductility when the alloy’s strength increased, commonly referred to as the “strength-ductility trade-off phenomenon” [19]. Although the uniaxial tensile tests were performed on AA and NA samples with comparable hardness levels, the ductility decrement ratios of AA and NA were significantly different. The elongation difference between AA and NA was verified by the strain hardening rate as a function of $\sigma -\sigma_{y}$, which was adopted to confirm the work hardening ability in AA (10 h) and NA (570 d), as shown in Figure 2c. The strain hardening rate (θ) was calculated based on the Kocks–Mecking (KM) plots (θ=dσ/dε) [20]. The θ value dramatically decreased in the initial stage due to the elastic-to-plastic deformation transition. Then, the θ value decreased continuously until failure. A larger θ value corresponded to uniform elongation and a delay of necking, indicating better ductility [21,22]. As shown in Figure 2c, the strain-hardening rate of NA (570 d) was higher than that of AA (10 h) during the entire plastic deformation process, which was responsible for its greater elongation at break.

### 3.2. Precipitate Analysis and Microstructure of Al–Mg–Zn Alloy Sheet after AA and NA

**Figure 3:**
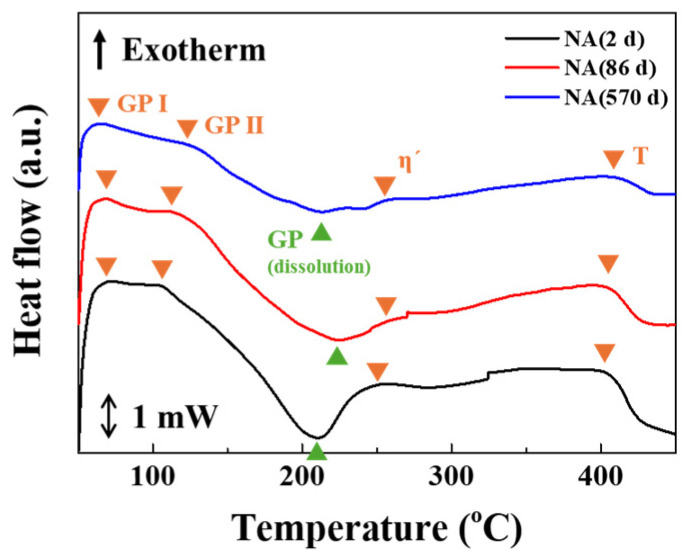
Thermal analysis of NA-treated Al–Mg–Zn alloy sheets using DSC with 10 °C/min heating rate.

Figure 3 shows the DSC curves of Al–Mg–Zn alloy sheets after aging treatment at a heating rate of 10 °C/min. The peaks in the curves indicate an exothermic reaction for the upward peak and an endothermic reaction for the downward peak; hence, the peak temperatures reflect the formation and dissolution of precipitates. Previously, the formation of precipitates in Al–Mg–Zn alloy after artificial aging was sufficiently examined [5,6,7,8,9]. The strengthening precipitates, η′ and η-phases, in peak artificially aged Al–Mg–Zn alloy were formed and distributed. Meanwhile, the main strengthening phases of the naturally aged alloy were not sufficiently discussed because it is hard to discover the clusters and precipitates of naturally aged alloy with microstructure analysis in the initial stage and specific time. Hence, it is necessary to define the dominant precipitates in NA-treated alloy samples using thermal analysis. Some researchers have revealed that the cluster and the GP zone were formed in the temperature range from 50 to 150 °C, and the GP zone was dissolved around 200–250 °C. Furthermore, η′, η, and T-phases could be developed gradually around 250–400 °C [23]. Above 200 °C, the dissolution of GP and clusters occurred with an endothermic peak, and then, η′ and η-phases were formed at the range of 250–300 °C. T-phases, which are stable phases, were formed after over-aging, at over 400 °C. These precipitation processes occurred at similar temperature ranges for naturally aged (NA) samples. At the initial stage, the peaks related to the formation of GP and clusters were observed in the temperature range of 50–150 °C, marked as GP I and GP II in NA (570 d). These thermal analyses indicate that solute clusters and the GP zone can still be formed in NA (570 d) through aging treatment, and the growth of solute clusters and the GP zone also occurred. Then, the GP zone decomposed at 200–230 °C. As seen in the DSC curves in Figure 3, the area, which is related to the formation of the GP zone, decreased according to the natural aging time increasing. The microstructure and compositional condition of NA (2 d) samples were close to the as-quenched (AQ) state; therefore, a large amount of solute clusters and GP zones can be formed. After that, η′-phases developed around 250 °C. From the DSC data, the dominant precipitates in naturally aged samples were composed of solute clusters, GP zones, and η′-phases. Especially, η′-phases might exist in the long-term aged samples, such as NA (86 d) and NA (570 d). The strengthening effect of those precipitates will be discussed.

The composition of the supersaturated solid solution in the matrix influences the potency of the precipitates in the alloy. From the peak temperature data of DSC analysis, the activation energy of each precipitate can be calculated using the Kissinger method in order to provide a thorough examination of precipitation in the AA- and NA-treated alloy sheets.

**Figure 4:**
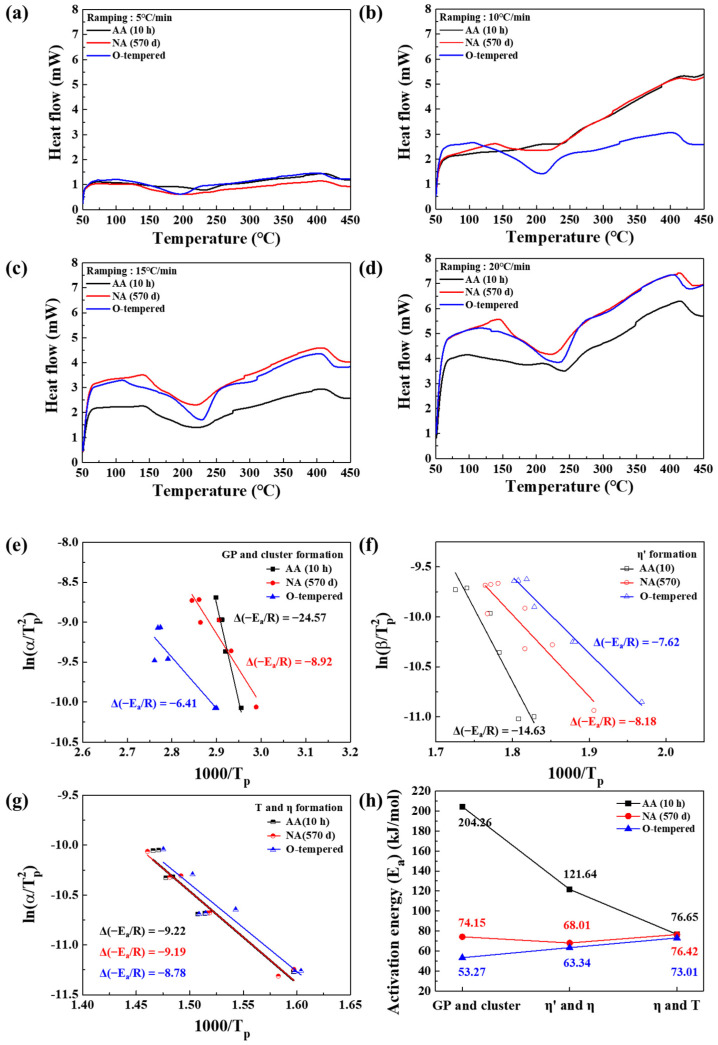
DSC analysis of AA-, NA-treated, and O-tempered Al–Mg–Zn alloy sheets with (**a**) 5, (**b**) 10, (**c**) 15, and (**d**) 20 °C/min heating rate. ln(α/(Tp^2^)) is a function of (1000/Tp) of (**e**) GP and solute clusters, (**f**) η′-phases, and (**g**) η and T-phases in AA-, NA-, and O-temper-treated Al–Mg–Zn alloys. (**h**) Activation energy calculated by Kissinger’s method.

Figure 4 shows the raw data of DSC analysis at different heating rates (5, 10, 15, and 20 °C/min), and their scattered points of the peak temperature were plotted. The activation energies of forming (e) GP zones, solute clusters, (f) η′-phases, and (g) η and T-phases in AA (10 h), NA (570 d), and the O-temper sample were calculated using Kissinger’s method, which is expressed as follows [24]:

(1) $$\ln\left(\frac{\alpha }{T_{p}^{2}}\right)=\left(\frac{{-E}_{a}}{R}\right)\left(\frac{1}{T_{p}}\right)+C$$

where *α*, *T_p_*, *E_a_*, *R*, and *C* represent the heating rate, peak temperature, activation energy, gas constant, and constant number, respectively. The relationship between $ln(\alpha /T_{p}^{2})$ and 1$/T_{p}$ allowed for the evaluation of the activation energy using the slope of the linear plot. As shown in Figure 4e,h, the activation energies of the GP and cluster formation for AA (10 h), NA (570 d), and O-temper were 204.26, 74.15, and 53.27 kJ/mol, respectively. The formation of the GP zone and solute clusters was more favorable in the O-temper state than in aged Al–Mg–Zn alloy sheets. Moreover, naturally aged alloy has slightly higher activation energy than O-temper alloy, so the formation peak of the GP zone appears in the DSC data, as shown in Figure 3. Furthermore, it is difficult for AA (10 h) to form GP zones and clusters because of their high formation energies. That result indicates that artificially aged alloy (AA (10 h)) had formed the precipitates using solute atoms, so these alloys include more developed precipitates than other samples. The activation energy for the formation of the η′-phase is evaluated with the same method. As shown in Figure 4f,h, O-tempered and NA (570 d) samples had a similar value of activation energy for the formation of η′-phases. On the other hand, though the activation energy for forming a precipitate decreased, AA (10 h) still had higher activation energy than other samples. The activation energies for forming T and η-phases have comparable values in the AA (10 h), NA (570 d), and O-tempered samples. Notably, the formation of T and η-phases in Al–Mg–Zn alloy sheets occurred in an over-aged state; hence, the initial aging condition did not influence the final aging process. From the thermal analysis of AA (10 h) and NA (570 d)-treated alloy sheets, we can predict the major strengthening precipitates of each aged Al–Mg–Zn alloy sheet. GP zones and solute clusters are still formable in the naturally aged samples; moreover, η′-phases can be formed in NA (570 d). However, the precipitate of AA (10 h) is already formed as stable phases because the formation energy for precipitates is higher than NA (570 d) and O-tempered alloy samples. As a result, these different precipitates could induce different interactions with dislocations, which are generated after plastic deformation; hence, the observation using TEM was conducted.

**Figure 5:**
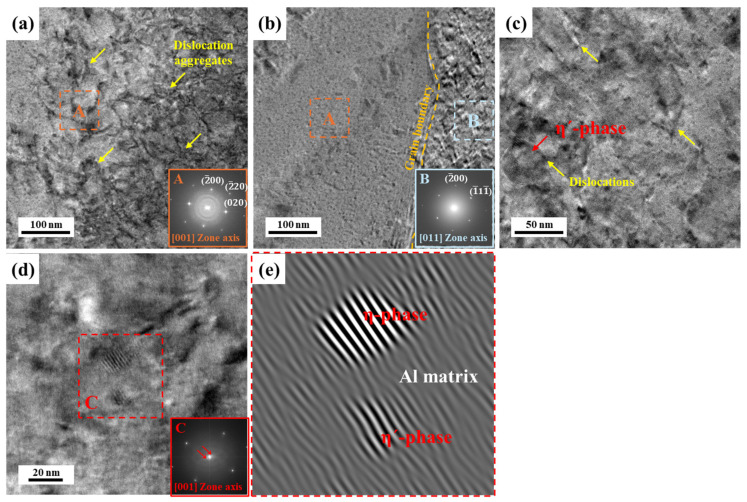
(**a**–**d**) HRTEM images and Fast Fourier Transform (FFT) patterns of AA (10 h) with Al (001) zone axis after 10% tensile deformation. (**e**) The corresponding inverse FFT (IFFT) images of area C with red line in the image (**d**). Yellow arrows indicate the dislocations, and red indicates the precipitates as shown.

Figure 5 shows the HRTEM images of 10% deformed AA (10 h) obtained along the (001) zone axis. As mentioned previously, AA (10 h) had a higher dislocation density than NA (570 d). The dislocations generated during deformation tangled, as shown in Figure 5a,c. However, the number of tangled dislocations decreased in the vicinity of grain boundaries, as shown in Figure 5b. The glide of deformation-induced dislocations is hindered by the precipitates in the matrix. As aforementioned in Figure 3 and Figure 4, the major strengthening precipitates of artificially aged Al–Mg–Zn alloy sheets are composed with the η′ and η-phases, and these precipitates are observed with the HRTEM and its corresponding IFFT images, as shown in Figure 5d,e. Moreover, in the FFT pattern, an extra spot is detected from the selected area C except for the aluminum matrix. Hence, the η′-phase with a semi-coherent interface and the η-phase with an incoherent interface are detected by the IFFT images. Uniformly distributed precipitates with semi-coherent or incoherent interfaces generally prevent the movement of dislocations; hence, dislocations agglomerate with precipitates.

**Figure 6:**
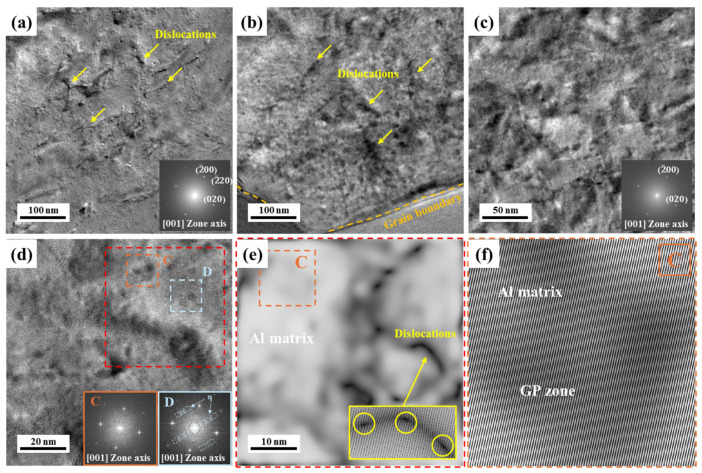
(**a**–**d**) HRTEM images and FFT patterns of NA (570 d) and with Al (001) zone axis after 10% tensile deformation. (**e**,**f**) The corresponding IFFT images of selected area in the image (**d**).

In contrast, NA (570 d) has fewer dislocations in the matrix, as shown in Figure 6a–c. Different from AA (10 h), a few dislocations existed near the grain boundaries in NA (570 d), as shown in Figure 6b. Furthermore, the number of tangled or agglomerated dislocations is fewer than the AA (10 h) sample even though those two samples have the same Vickers hardness level; hence, we can estimate that the strengthening precipitates of NA and AA are different. As aforementioned in the thermal analysis, the NA (570 d) sample can have solute clusters, GP zones, and η′-phases. The HRTEM images do not show the distinct precipitate because of its relatively small size and unclear interface with a matrix. Furthermore, the extra spots for precipitates are not defined from the FFT patterns of the selected area, as shown in Figure 6a,c,d at the C area. Solute clusters and GP zones in Al–Mg–Zn alloys have been detected by Zhuang et al. with HRTEM and corresponding IFFT imaging methods [15]. Figure 6d also shows the small clusters in area C, and their corresponding FFT pattern is consistent with the FFT results of the matrix in Figure 6a,c. Furthermore, IFFT images indicate that these small particles have coherent interfaces with the matrix; hence, they could be treated as a GP zone after natural aging. IFFT images in Figure 6e also show the dislocations. The formation of an η′-phase is uncommon in the early stages of natural aging; however, the NA (570 d) sample experienced a long time to form a η′-phase, and it is defined with the extra spots in the FFT pattern of area D in Figure 6d [25]. The naturally aged NA (570 d) sample has a variety of precipitates to strengthen the alloy; however, the artificially aged AA (10 h) sample is dominantly composed of η′ and η-phases. As a result, the deformation behaviors via the movement of dislocations are varied with the type of aging process because the movement of dislocation during plastic deformation can be affected by precipitates.

**Figure 7:**
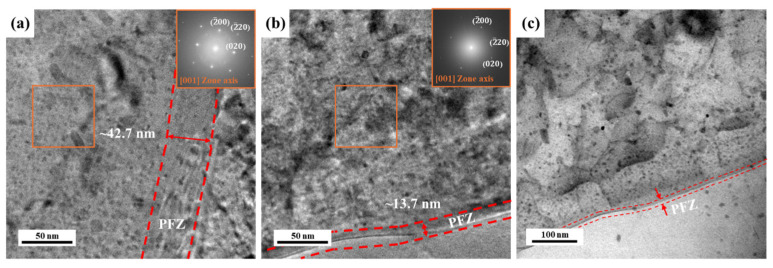
HRTEM images of (**a**) AA (10 h) and (**b**) NA (570 d) at the vicinity of grain boundaries. (**c**) Annular bright field (ABF) images of NA (570 d) with STEM observations.

In terms of deformation, the precipitate distributions and dislocation densities were significantly different near the grain boundaries. This is a well-known phenomenon—the precipitate-free zone (PFZ)—developed by the interaction between the diffusion of solute elements and the vacancies at the grain boundaries [26]. Studies on PFZ are beneficial for understanding the effects of aging on the mechanical properties of alloy sheets. A comparison of the microstructures of AA (10 h) and NA (570 d) revealed that the PFZ in AA (10 h) was wider than that in NA (570 d), as shown in Figure 7. The thickness of PFZ is closely related to the mechanical properties of alloys; hence, studies on PFZ are important to understand the effects of precipitation-hardening processes on the mechanical properties of alloy sheets [27,28,29,30].

The PFZ thicknesses in AA (10 h) and NA (570 d) were approximately 42.7 and 13.7 nm, respectively, as shown in Figure 7. AA (10 h) had a wider PFZ area with more distributed precipitates than NA (570 d). Previous studies revealed that the formation of PFZ is affected by the depletion of vacancy and solute atoms in the early and later stages of aging. The width of PFZ is firstly decreased in the early stage of aging and then gradually increased with aging time at 433 K [31]. Hence, the width of PFZ can be changed with the aging condition and diffusion rate. Using plasticity simulations, Kdadyko et al. examined the work hardening scenario in the PFZ area; the local strain was strongly correlated with deformation when perfect PFZ plasticity was assumed [32,33]. Thus, AA (10 h) with a larger PFZ area induced more local strains in the PFZ area owing to the absence of solute elements and precipitation. On the other hand, the work hardening in the PFZ of NA (570 d) showed similar behaviors to those of the bulk alloy samples, which was attributed to its small PFZ area, as shown in Figure 7b,c. NA (570 d) had solute clusters and a GP zone as the strengthening phases, which were difficult to observe in bright-field TEM images. For these reasons, the annular bright field (ABF) STEM image can help define solute clusters using the brightness contrast based on their atomic weight. The atomic weight of Zn is higher than those of Al and Mg, and the uniformly distributed strengthening phases show a darker contrast, as shown in Figure 7c.

Furthermore, the PFZ thickness can affect the localization of the plastic deformation. It is commonly accepted that as the PFZ thickness increases, the restriction from the surrounding grains diminishes, and the localization of plasticity increases. Therefore, the mechanical properties of an aluminum alloy with a PFZ can be expressed by the following equation [34]:

(2) $$\sigma =\sigma_{0}+\tau_{c}{\left(\frac{D\varepsilon }{6b}\right)}^{\frac{1}{2}}\times (1+\frac{\lambda }{W_{0}})$$

where *σ*, *σ*_0_, *τ_c_*, *D*, *ε*, *b*, *λ*, and *W*_0_ stand for the stress, frictional stress, critical shear stress for cross slip, average grain diameter, strain of sample, Burgers vector, interparticle spacing, and width of PFZ, respectively. The total strain of the specimen is related to the work hardening rate; thus, the hardening rate can be obtained in a differentiated form of Equation (2):

(3) $$\frac{d\sigma }{d\varepsilon }=aW_{0}^{-1}+b$$

According to this equation, the hardening rate decreases as the thickness of the PFZ increases. The hardening rate of AA (10 h) is lower than that of NA (570 d), as shown in Figure 2c. Furthermore, an increase in PFZ thickness may stimulate the formation of grain boundary precipitates and create voids, which is favorable for fracture propagation along the grain boundaries.

The above observations on Al–Mg–Zn alloy sheets provide insights into designing high-ductility and high-strength alloys with age-condition control. Zhang et al. [20] studied the positive effect of plastic deformation in naturally aged Al–Zn–Mg alloys using experimental and calculation methods. Solute clusters, formed after long-term NA, can be dissolved at the initial deformation stage because they improve strain hardening ability. Future research should focus on investigating the correlation between precipitation and deformation in these alloy types with the aim of engineering microstructures with enhanced properties.

### 3.3. Anisotropy Analysis of Al–Mg–Zn Alloy Sheets after AA and NA

**Figure 8:**
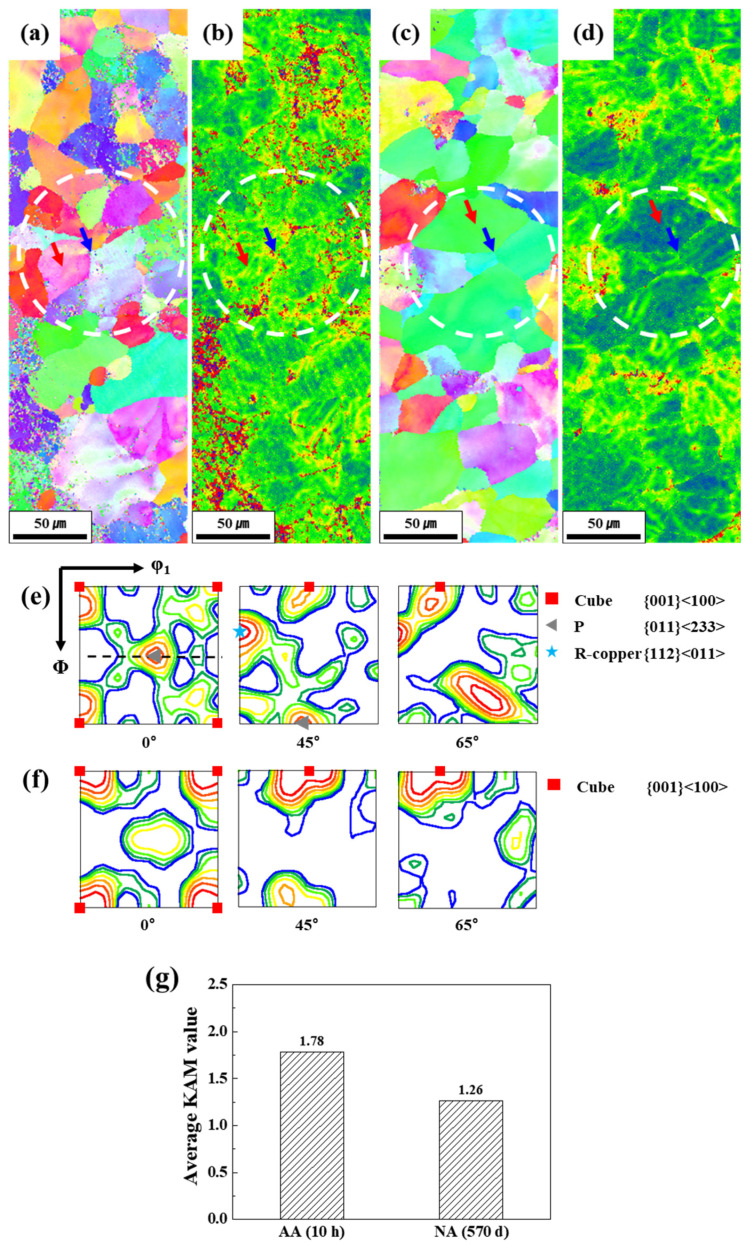
EBSD images and kernel average misorientation (KAM) map of (**a**,**b**) AA (10 h) and (**c**,**d**) NA (570 d) after 10% deformation. The white circle indicates the difference in stress concentration. Texture evaluation with (orientation distribution function) ODF for (**e**) AA (10 h) and (**f**) NA (570 d). (**g**) Graph of the average KAM value of AA (10 h) and NA (570 d). And the red and blue arrows indicate the internal and boundaries of grain.

Enhanced strain hardening ability and elongation to fracture, associated with delay of necking, could exhibit a reasonable strength and large uniform elongation; hence, NA with a higher strain hardening rate (θ) value could accumulate a larger density of dislocation in the alloy than AA. The characteristics according to the aging condition in mechanical properties are investigated via EBSD methods after 10% tensile deformation. Plastically deformed polycrystalline materials contain dislocations in their matrices, as shown in the TEM images in Figure 5, Figure 6 and Figure 7. Deformation-induced dislocations can be classified into geometrically necessary dislocations (GNDs) and statistically stored dislocations (SSDs) [35,36,37]. Generally, GNDs’ density is related to microstructure characteristics, such as grain size and texture. The density of the GNDs can be evaluated through EBSD misorientation, which is expressed as follows [38]:

(4) $$\rho_{GNDs}=\frac{\beta \times \varphi }{b\times ∆x}$$

where *β* is a constant determined by the type of dislocation boundaries, *φ* is the average misorientation within a certain distance Δ*x*, and *b* is the Burgers vector. With the increase in GND density upon deformation, the kernel average misorientation (KAM) value, i.e., θ, also increases, as calculated using Equation (4) [39]. As aforementioned above in TEM observations, GNDs are generated in the internal region of grains; however, it is hard to visualize the distribution of GNDs. Consequently, the distribution of dislocation after plastic deformation was evaluated using a KAM map. Figure 8a–d shows the inverse pole figure EBSD images and KAM maps of AA (10 h) and NA (570 d) after 10% deformation. The dislocation density can be evaluated from the intensity of KAM. The average KAM values of AA (10 h) and NA (570 d) were calculated as approximately 1.78 and 1.25, respectively, using the implemented source of the EBSD analysis program, as shown in Figure 8g. Although the same amount of plastic deformation was applied to the samples, AA (10 h) exhibited a higher dislocation density than NA (570 d). Red and blue arrows indicate the internal and boundary of grains, respectively. In the white circle in the KAM map of AA (10 h), a high density of dislocations is accumulated, as shown in Figure 8a,b. Furthermore, dislocations are accumulated in the internal area of grains, marked as red and blue arrows; hence, the grain boundaries were not revealed clearly. However, the density of GNDs is lower in the NA (570 d) grains than in the AA (10 h) grains, as marked by the white circle in Figure 8c,d. The average density of GND in NA (570 d) is lower than AA; moreover, the partial region has a lower KAM value in the internal grain than the average value, as shown with red and blue arrows in Figure 8c,d. The evaluation of EBSD results shows the same dislocation evaluations as the TEM observations. Notably, the density of GNDs in NA (570 d) was only slightly lower and can be more uniformly distributed in grains than in AA (10 h) at the same strain level; hence, the NA could accumulate more GNDs, contributing to higher elongation. Furthermore, the crystalline order of the plastically deformed Al alloy developed in a favorable texture along the deformation direction. In the rolled Al alloy sheets, Cube, Goss, and P textures developed initially after O-temper treatment [40]. Furthermore, Cube, Goss, and P textures were formed as fibers when the Al alloy was anisotropically deformed. As shown in Figure 8e,f, Cube, P, and rotated-copper textures were mainly developed in the 10%-deformed AA (10 h) sample, whereas only Cube textures were developed in the NA (570 d) sample.

**Figure 9:**
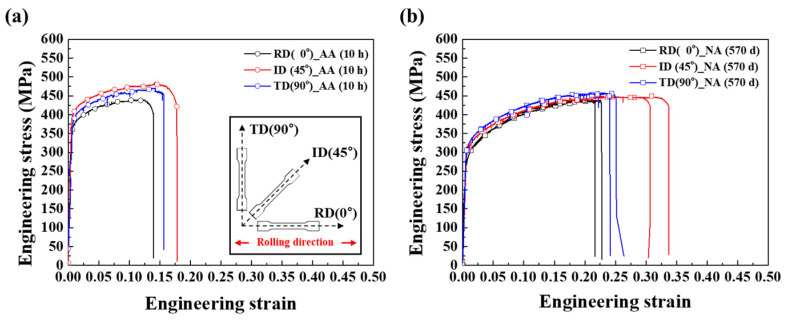
Stress–strain curves of (**a**) AA (10 h) and (**b**) NA (570 d) measured at different angles to the rolling direction (0°, 45°, and 90°).

Based on the above mechanical properties and precipitation analysis, aluminum alloy can exhibit different deformation behaviors depending on the aging temperature. In particular, the generation and accumulation of dislocations in plastic deformation appear nonuniform; hence, the anisotropy in plastic deformation was measured by tensile tests along the three directions. Figure 9 shows the stress–strain curves obtained for AA (10 h) and NA (570 d) at different angles to the rolling direction: 0° (RD), 45° (ID), and 90° (TD). The results for each sample showed that the YS and UTS of the NA samples were comparable. However, the AA sample tested in the ID direction possessed higher strengths than those tested in the RD and TD directions. The largest difference was observed between the ID and RD. Furthermore, the elongation at break tended to increase in ID and TD compared to RD. The R-value is defined as the ratio between the strain along the thickness and width, suggesting that a lower R-value implies excessive deformation along the thickness direction. As shown in Table 2, the R-value of the ID direction is always higher than other directions; hence, the ID direction can exhibit the highest elongation value. From the results above, though the yield strength of AA has a higher value than those of the NA sample, NA shows better ductility and comparable strength than AA because of the different strengths and elongations required for fracture along the directions [41]. To estimate the anisotropic deformation, the anisotropic coefficient (R) for the AA (10 h) and NA (570 d) samples are listed in Table 2 according to the tensile test direction and following this equation [42]:

(5) R−value=∆wwi∆tti

where ∆w and ∆t stand for the change of width and thickness after tensile test. $w_{i}$ and $t_{i}$ stand for the initial width and thickness of tensile specimen (ASTM E8). It should be stressed that the anisotropic coefficient, R-value, for an isotropic material is 1; R > 1, which means that the material is resistant to thinning, while R < 1 implies that the material will be reduced more in thickness than in width [42]. The R-value of NA (570 d) has a higher value than the AA (10 h) sample. Notably, AA sheets exhibit significant anisotropy in strength and plasticity compared to NA sheets. These results indicate that the precipitates in Al–Mg–Zn alloy sheets exhibit anisotropy during deformation, depending on the type of precipitates.

## 4. Conclusions

This study discovered that NA-treated Al–Zn–Mg alloys exhibited better ductility than AA-treated alloys; despite the same strength level, NA-treated samples exhibited about 10% longer elongation at break than their AA-treated counterparts. The capability of dislocation accumulation in the grain interior of the NA samples resulted in a higher strain-hardening rate and, consequently, higher ductility. After deformation at the same stress level, the densities of the GNDs in AA (10 h) and NA (570 d) were only marginally different. Furthermore, NA (570 d) is more favorable to deformation because of a higher anisotropic coefficient than AA (10 h), which contributed to the uniform elongation.

(1) During the aging process, the Vickers hardness of Al–Mg–Zn alloy sheets was increased regardless of aging conditions. In the uniaxial tensile test, however, artificially aged sheets exhibited about 15% elongation to fracture, whereas naturally aged sheets showed about 25% elongation to fracture property.
(2) The analysis of precipitates in AA (10 h) and NA (570 d) samples was performed using the DSC, and their activation energy to form the precipitates were calculated with the Kissinger method. The AA (10 h) sample dominantly had η′ and η-phases as strengthening precipitates; however, NA (570 d) samples had solute cluster, GP, and η′-phases.
(3) TEM observation indicated that the densities of the GNDs in NA (570 d) were lower than AA (10 h); however, they were distributed more uniformly both internal and boundaries of grains. Furthermore, PFZ width varied depending on the aging conditions; hence, NA-treated alloy had a narrower PFZ width than AA-treated alloy.
(4) The NA-treated sheets exhibited notable anisotropy in strength and plasticity with a higher average R-value than their AA-treated counterparts.

## Figures and Tables

**Table 1:** Chemical composition of Al–Mg–Zn alloy (wt. %).

	Mg	Zn	Mn	Fe	Al
Desired composition	5	4	0.3	-	Bal.
Measured composition	4.85	3.97	0.27	0.08	Bal.

**Table 2:** Anisotropic coefficient (R) for AA (10 h) and NA (570 d) according to the tensile test direction.

Aging Condition	Direction	Anisotropic Coefficient (R)
AA (10 h)	0°	0.602
	45°	0.672
	90°	0.619
NA (570 d)	0°	0.778
	45°	0.828
	90°	0.698

## Data Availability

Data are available in a publicly accessible repository.
